# Clinical Characteristics and Long-Term Prognosis of Colorectal Mucosa-Associated Lymphoid Tissue Lymphoma According to the Endoscopic Classification and Treatment Modality: A Multicenter Study

**DOI:** 10.3390/cancers17050750

**Published:** 2025-02-22

**Authors:** Seung Min Hong, Dong Hoon Baek, Geun Am Song, Hong Sub Lee, Seung Bum Lee, Ra Ri Cha, Tae-Oh Kim, Jae Hyun Kim, Jong Hoon Lee, Busan Ulsan Gyeongnam Intestinal Study Group Society (BIGS)

**Affiliations:** 1 Department of Internal Medicine, Pusan National University School of Medicine and Biomedical Research Institute, Pusan National University Hospital, Busan 49241, Republic of Korea; lucky77i@naver.com (S.M.H.); gasong@pusan.ac.kr (G.A.S.); 2 Department of Internal Medicine, Busan Paik Hospital, Inje University College of Medicine, Busan 47392, Republic of Korea; epoch0123@naver.com; 3 Department of Gastroenterology, Ulsan University Hospital, University of Ulsan College of Medicine, Ulsan 44033, Republic of Korea; sblee@uuh.ulsan.kr; 4 Department of Internal Medicine, Gyeongsang National University Changwon Hospital, Gyeongsang National University School of Medicine, Changwon 51472, Republic of Korea; rari83@naver.com; 5 Department of Internal Medicine, Inje University, Haeundae Paik Hospital, Busan 48108, Republic of Korea; kto0440@paik.ac.kr; 6 Department of Gastroenterology, Kosin University College of Medicine, Busan 49267, Republic of Korea; kjh8517@hanmail.net; 7 Division of Gastroenterology, Department of Internal Medicine, Dong-A University College of Medicine, Busan 49201, Republic of Korea; jh2002@dau.ac.kr

**Keywords:** mucosa-associated lymphoid tissue lymphoma, colorectum, treatment, endoscopic type

## Abstract

**Background/Objectives**: The clinical characteristics of colorectal mucosa-associated lymphoid tissue (MALT) lymphoma remain poorly defined, and there is no standardized treatment for the disease. Therefore, we investigated the clinical characteristics of colorectal MALT lymphoma and its prognosis based on different treatment modalities. **Methods**: A retrospective analysis was performed on patients diagnosed with colorectal MALT lymphoma from 2003 to 2021 across six hospitals in Korea’s Busan–Ulsan–Gyeongnam area. Macroscopic findings classified all cases into polyposis type, mass-forming type, subepithelial lesion type, and inflammatory type. **Results**: Fifty-one patients were enrolled. The median age was 59 years, and 27 patients (52.9%) were male. Five patients (9.8%) were stage IV at initial diagnosis. As for the endoscopic type, the polyposis type was the most common (39.2%). There was no statistically significant difference in disease progression according to the endoscopic type (*p* = 0.813). Three cases of disease progression were confirmed in stage I after treatment, and one of them died due to disease progression. No disease progression was identified in other stages. According to the treatment modality, disease progression was identified in 1 of 16 patients who underwent endoscopic resection and 2 of 16 patients who underwent chemotherapy. There was no disease progression in the observation group. However, there was no statistically significant difference in disease progression according to treatment modality (*p* = 0.889). **Conclusions**: Colorectal MALT lymphoma showed good prognosis regardless of the initial stage, endoscopic type, or treatment modality.

## Simple Summary

Colorectal mucosa-associated lymphoid tissue (MALT) lymphoma is a rare subtype of non-Hodgkin’s lymphoma. Unlike gastric MALT lymphoma, which has a standardized treatment approach, colorectal MALT lymphoma lacks an established therapeutic approach. This study analyzed the clinical characteristics and long-term outcomes of 51 patients diagnosed with colorectal MALT lymphoma across six hospitals in Korea’s Busan–Ulsan–Gyeongnam area. The findings revealed that the disease was typically detected at an early stage, progressed slowly, and had an excellent prognosis with high survival rates. Treatment methods were not associated with disease progression rates. All disease progression occurred at stage I, showing that advanced stages were not necessarily related to poor prognosis. Additionally, none of the nine patients who underwent observation without treatment experienced disease progression. These results underscore the indolent nature of colorectal MALT lymphoma and provide valuable insights to guide individualized management strategies.

## 1. Introduction

Mucosa-associated lymphoid tissue (MALT) lymphoma, first described by Isaacson and Wright in 1983, is a distinct subtype of non-Hodgkin’s lymphoma (NHL) [1,2]. As the third most common form of NHL, MALT lymphoma accounts for approximately 5–8% of all NHL cases, with the gastrointestinal (GI) tract being a frequent site of involvement [3,4].

Within the spectrum of GI MALT lymphoma, the stomach is the most commonly affected organ, followed by the colorectum and small bowel [5,6]. However, colorectal MALT lymphoma remains a relatively rare entity, comprising only 2.5% of all MALT lymphomas [7,8,9,10,11]. This rarity is exemplified by the low incidence rates reported in various studies, such as the Canada-based study that identified only six cases over a decade in a population of 1.3 million [12].

The pathogenesis of MALT lymphomas vary depending on the site of origin. *Helicobacter pylori* infection and subsequent chronic inflammation play a well-established role in gastric MALT lymphoma [13]. Similarly, *Campylobacter jejuni* has been implicated in small-intestinal MALT lymphoma [5]. However, the etiology of colorectal MALT lymphoma remains elusive, with no clear infectious agent identified to date [14].

The clinical presentation and endoscopic features of colorectal MALT lymphoma are diverse and non-specific, often mimicking other colorectal pathologies. This variability poses significant challenges in diagnosis and management. Unlike gastric MALT lymphoma, where *H. pylori* eradication is often the first-line treatment, there is no standardized therapeutic approach for colorectal MALT lymphoma. Treatment options include endoscopic resection, chemotherapy, radiation therapy, or surgical resection, but the optimal strategy remains unclear [7,9,15,16].

Given the rarity of colorectal MALT lymphoma and the lack of large-scale studies, there is a pressing need for comprehensive research to better understand its clinical characteristics, prognostic factors, and optimal treatment strategies. This study aimed to address this knowledge gap by investigating the clinical features and long-term prognosis of colorectal MALT lymphoma in the Busan–Ulsan–Gyeongnam area of Korea.

## 2. Materials and Methods

### 2.1. Patients

Patients diagnosed with colorectal MALT lymphoma between October 2003 and May 2021 at six referral hospitals in the Busan, Ulsan, and Gyeongnam regions of South Korea (Pusan National University Hospital, Inje University Busan Paik Hospital, Inje University Haeundae Paik Hospital, Ulsan University Hospital, Gyeongsang National University Changwon Hospital, and Kosin University Gospel Hospital) were included in this study. All patients were diagnosed through histopathological examinations performed by GI pathology specialists at each hospital. A total of 51 patients were ultimately included in the study. This research was approved by the Institutional Review Board (IRB) of our institution (IRB No. 2201-007-111).

### 2.2. Data Collection and Definitions

The medical records of 51 patients included in the study were retrospectively analyzed. Baseline characteristics such as age, sex, and underlying diseases were examined. Additionally, the presence of B symptoms at the time of diagnosis, levels of lactate dehydrogenase (LDH), beta-2-microglobulin (B2MG), and *H. pylori* infection status were assessed. Disease-related characteristics, including the initial stage, lesion location, lesion multiplicity, first endoscopic impression, and endoscopic type, were also investigated. B symptoms were defined as unexplained fever exceeding 38 °C, night sweats, and unexplained weight loss of more than 10% within the past 6 months [17]. MALT lymphoma is an indolent extranodal lymphoma composed of heterogeneous small B cells. The lesions in this study were all located in the terminal ileum or colorectum, and specimens were obtained through colonoscopic biopsy, colonoscopic resection, or surgical resection. Furthermore, the collected specimens were evaluated by GI pathology specialists, and immunohistochemistry tests were performed to confirm the diagnosis. Positive markers for MALT lymphoma include CD20, CD79a, and BCL-2, while negative markers include CD5, CD10, cyclin D1, and BCL-6 [5,10,18].

**Figure 1:**
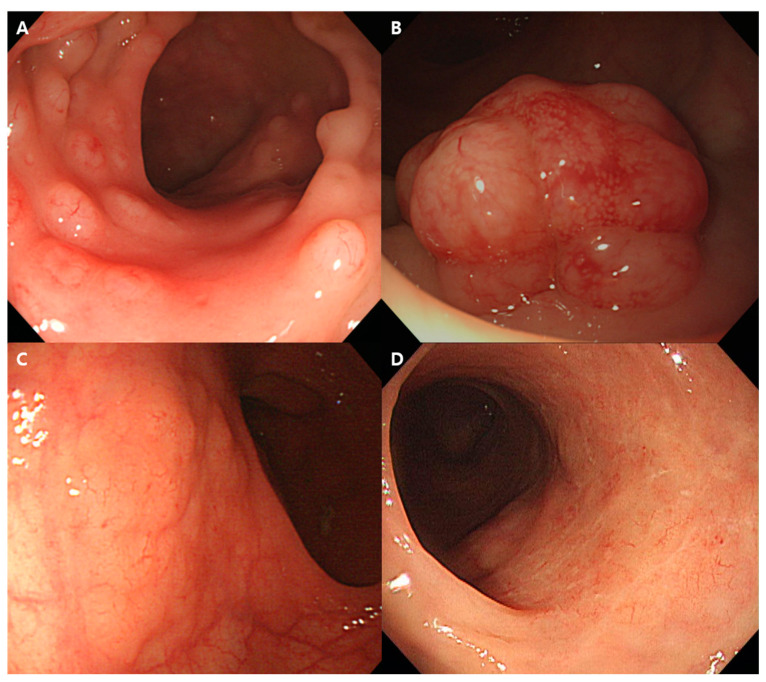
Four endoscopic classifications of colorectal MALT lymphoma. (**A**) polyposis type, (**B**) mass-forming type, (**C**) subepithelial lesion type, and (**D**) inflammatory type.

Disease staging was conducted using the Lugano classification system for GI MALT lymphoma [19,20]. The endoscopic type was classified into four categories: polyposis type, mass-forming type, subepithelial lesion type, and inflammatory type. The polyposis type was defined as the presence of more than five polypoid lesions, each smaller than 15 mm, within at least one colonic segment, regardless of whether lesions are present in multiple segments. The mass-forming type was characterized by lesions protruding into the intestinal lumen, with a surface showing redness and frequently enlarged vessels, and was occasionally accompanied by ulceration or erosion. The subepithelial lesion type referred to elevated lesions that appeared to have subepithelial components, generally flat with a tendency to spread laterally rather than protruding into the lumen, often featuring a smooth surface with occasional enlarged vascular patterns. The inflammatory type was defined as lesions that did not fit into the other three categories and primarily presented with mucosal redness with occasional enlarged vascular patterns, multiple erosions, or ulceration (Figure 1).

Treatment methods received by the patients were investigated, including endoscopic resection, surgical resection, radiation therapy, chemotherapy, combinations of multiple treatment modalities, and observation. Differences in disease progression based on the treatment methods were analyzed. Additionally, the initial stage and the occurrence of disease progression were analyzed based on the endoscopic type. Disease progression was defined as confirmed disease progression, relapse, or patient death. Based on the time of diagnosis, overall survival was defined as the duration until patient death, progression-free survival as the duration until disease progression or patient death, and disease-specific survival as the duration until death caused by colorectal MALT lymphoma.

### 2.3. Statistical Analyses

The patients’ ages were expressed as median values with interquartile range (IQR), and other categorical variables were presented as numbers and simple percentages. Statistical significance was evaluated using the χ^2^ test or Fisher’s exact test for categorical variables. A *p*-value of less than 0.05 was considered statistically significant. Survival analysis was performed using the Kaplan–Meier method. All statistical analyses were conducted using SPSS software (IBM^®^ SPSS^®^ Statistics for Windows, Version 25.0; IBM, Chicago, IL, USA).

## 3. Results

### 3.1. Clinical Characteristics at Diagnosis

The median age of the patients was 59 years, with 27 of them (52.9%) being male. A total of 47 patients (92.2%) had no underlying diseases, while one patient each was undergoing treatment or follow-up for liver cirrhosis, rheumatoid arthritis, pulmonary tuberculosis, or pituitary adenoma. Only one patient reported B symptoms. At the time of diagnosis, elevated LDH levels were observed in 8 patients (15.7%), and elevated B2MG levels were noted in 18 patients (35.3%). *H. pylori* infection was confirmed in 9 patients (17.6%) during the initial evaluation, while 25 patients (49.0%) had not undergone testing for *H. pylori* infection. Most patients (*n* = 40, 78.4%) were diagnosed at stage I, with only 5 patients (9.8%) at stage IV, and 2 (3.9%) and 4 (7.8%) patients diagnosed at stage II and IIE, respectively. Among the stage IV patients, bone marrow involvement was identified in two cases (Table 1).

### 3.2. Endoscopic Findings and Treatment Methods at Diagnosis

The most common lesion site was the rectum (*n* = 20, 39.2%), followed by the terminal ileum (*n* = 5, 9.8%), cecum (*n* = 4, 7.8%), and ascending colon (*n* = 5, 9.8%) within the right colon, including the transverse colon (*n* = 2, 3.9%). The left colon, comprising the descending colon (*n* = 4, 7.8%) and sigmoid colon (*n* = 1, 2.0%), had relatively fewer lesions. Lesions involving multiple segments were observed in 10 patients (19.6%). Among the three endoscopic types, excluding the inflammatory type, multiplicity was observed in 32 patients (66.7%). The most common endoscopic type was the polyposis type (*n* = 20, 39.2%), followed by the subepithelial lesion type (*n* = 15, 29.4%), mass-forming type (*n* = 13, 25.5%), and inflammatory type (*n* = 3, 5.9%).

The initial endoscopic impression was most frequently lymphoma (*n* = 27, 52.9%), followed by colon polyp (*n* = 13, 25.5%). Other impressions included neuroendocrine tumor (*n* = 6, 11.8%), colon cancer (*n* = 1, 2.0%), and lymphoid follicle (*n* = 2, 3.9%). Inflammatory diseases were also mistaken for lesions in some cases, including ulcerative colitis and tuberculosis colitis, with one case each.

Regarding treatment methods, endoscopic resection and chemotherapy were the most common, each performed in 16 patients (31.4%). Radiation therapy (*n* = 5, 9.8%) and surgical resection (*n* = 1, 2.0%) followed. Combination therapy was provided to four patients (7.8%), with two patients undergoing both endoscopic resection and chemotherapy and another two receiving endoscopic resection and radiation therapy. Furthermore, nine patients (17.6%) underwent observation and follow-up without specific treatment (Table 2).

### 3.3. Clinical Outcomes

Progression rates were analyzed to evaluate the prognosis based on the endoscopic type. There were two cases of progression in the polyposis type and one case in the subepithelial lesion type. However, these findings were not statistically significant (*p* = 0.813). Similarly, the initial stage showed no significant differences among the endoscopic types (*p* = 0.136) (Table 3). When progression rates were analyzed according to treatment methods, progression occurred in one patient who underwent endoscopic resection and in two patients who received chemotherapy, but this also did not show statistical significance (*p* = 0.889) (Table 4).

All cases of progression occurred at stage I. Two patients died during the follow-up period, one from disease progression and the other from sepsis due to severe infection. The patient who died from disease progression had initially achieved complete remission following R-CVP (rituximab, cyclophosphamide, vincristine, prednisone) as the first-line treatment but experienced relapse after 33 months. The patient continued additional chemotherapy (rituximab plus bendamustine), but the treatment was eventually discontinued due to a decline in general condition, leading to death from disease progression. Among the other two patients with progression, one had received R-CVP as the first-line treatment. Progression was confirmed after 41 months, and the patient underwent additional chemotherapy (rituximab plus bendamustine), maintaining stable disease. Subsequently, at the patient’s request, no further chemotherapy was administered, and the patient survived the follow-up period without additional progression. The other patient underwent first-line treatment with endoscopic submucosal dissection for a 2.5-cm subepithelial lesion type MALT lymphoma located in the descending colon. During the procedure, no residual lesion was seen macroscopically, and histological evaluation confirmed clear resection margins. However, follow-up examinations revealed relapse, and the patient subsequently underwent R-CVP as a second-line treatment, resulting in complete remission. The patient remained in remission without further relapses during the follow-up period.

**Figure 2:**
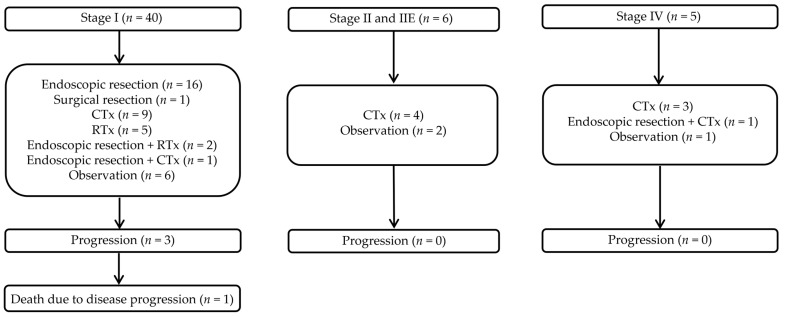
Progression and disease-related mortality according to initial stage. RTx, radiation therapy; CTx, chemotherapy.

Table 5 summarizes the clinical characteristics and clinical course of the three patients who experienced progression. Figure 2 illustrates the treatment methods used for each initial stage and their corresponding progression and disease-related mortality cases. Table S1 details the clinical characteristics of the nine patients who underwent observation without specific treatment, and none of them experienced progression.

**Figure 3:**
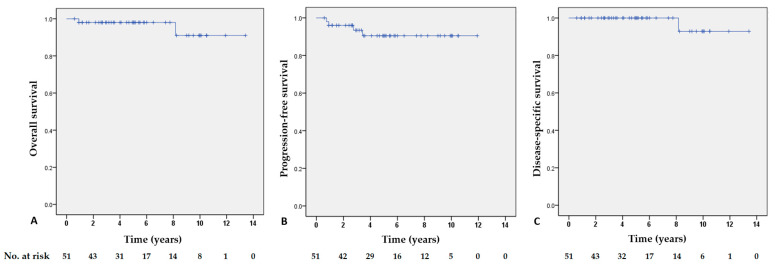
Patients’ survival rates. (**A**) overall survival, (**B**) progression-free survival, and (**C**) disease-specific survival. No., number.

Survival analysis revealed a 5-year overall survival rate of 98.0%, a 5-year progression-free survival rate of 90.5%, and a 5-year disease-specific survival rate of 100%. The 10-year overall survival rate was 91.0%, the 10-year progression-free survival rate was 90.5%, and the 10-year disease-specific survival rate was 92.9% (Figure 3).

## 4. Discussion

This study investigated the clinical characteristics and long-term prognosis of colorectal MALT lymphoma. It demonstrated the indolent nature of colorectal MALT lymphoma, with progression being rare or occurring after a prolonged period, and an excellent long-term prognosis. The 5-year overall survival and progression-free survival rates were 98.0% and 90.5%, respectively, and the 5-year disease-specific survival rate was 100%. While there was one case of death due to disease progression, this occurred 98 months after the initial diagnosis. In our study, neither the endoscopic type nor the treatment method showed significant associations with prognosis. Furthermore, no correlation was observed between the advanced stage and disease progression. To the best of our knowledge, this is the first multicenter study to investigate the clinical characteristics and long-term prognosis of colorectal MALT lymphoma.

The median age of the patients included in our study was 59 years, with an almost equal male-to-female ratio. The most common lesion site was the rectum, followed by the right colon, which included the terminal ileum. Additionally, 78.4% of the patients were diagnosed at stage I, and 11.7% were diagnosed at stage II or IIE, indicating that most patients were diagnosed at an early stage. Overall, these findings are consistent with those reported in previous studies [8,9,10,15,21,22,23,24,25]. In a retrospective study by Jeon et al., 73% of patients were diagnosed at stage I. Similarly, Kim et al. analyzed eight cases of colorectal MALT lymphoma reported in Korean literature and found that, among the five cases with recorded staging, four patients were classified as stage IE according to the Ann Arbor staging system [10,25]. This suggests that MALT lymphoma is generally diagnosed at an early stage. In addition, Maeshima et al. reported that among 467 patients with MALT lymphoma, 384 (82%) were diagnosed at stage I [26]. The fact that the disease is predominantly diagnosed at an early stage has two significant implications. First, when diagnosed early, patients are more likely to receive less invasive modalities such as endoscopic resection or radiation therapy instead of extensive surgical resection, which may require removal of a large intestinal segment. This treatment approach is directly associated with a better prognosis. Second, the predominance of early-stage diagnoses indicates that disease progression to an advanced stage takes a prolonged period, further supporting the indolent nature of MALT lymphoma. This indolent characteristic of MALT lymphoma likely explains why patients in our study who underwent observation alone maintained a favorable prognosis without progression.

GI MALT lymphoma is known to have a favorable prognosis. A relatively recent nationwide multicenter study conducted in Korea on the long-term clinical outcomes of gastric MALT lymphoma showed that most patients were diagnosed at an early localized stage, with excellent overall survival of 99.1% [27]. However, the incidence of colorectal MALT lymphoma is very low, leading to limited research. Most of the existing literature consists of case reports or case series, with a few literature reviews summarizing these cases [7,9,25]. Recently, a retrospective study from a single institution was published [10]. However, even when combining the patients from these studies, the numbers remain insufficient to fully understand the clinical characteristics of colorectal MALT lymphoma.

According to the currently available literature, colorectal MALT lymphoma displays diverse clinical characteristics, and, as no standardized treatment has been established, individualized approaches are required for each patient [15,28,29,30]. In our study, treatment was tailored to each patient’s specific situation, considering the location, morphology, multiplicity, and stage of the lesions. Chemotherapy was primarily considered in cases with advanced disease, such as stage IV, or when locoregional treatment was not feasible. Lesions with well-defined margins and a limited number of lesions were the primary targets for endoscopic resection. Radiation therapy and surgical resection were also considered as locoregional treatments, particularly for lesions that could not be treated with endoscopic resection.

Among the patients included in our study, nine patients (17.6%) underwent observation and follow-up without specific treatment. Interestingly, disease progression was not observed in patients who were managed with observation but rather in those who underwent endoscopic resection or chemotherapy. Similarly, Jeon et al. reported that none of the 5 patients who were observed without treatment among a cohort of 51 patients experienced progression [10]. In practice, factors such as old age, comorbidities, or patient refusal can prevent active treatment. Our findings suggest that observation and close monitoring can be an alternative for clinicians in such situations. However, due to the relatively small number of patients in our study, these results alone cannot support active recommendations for observation. Additionally, clinicians must carefully balance the risk of disease progression with the potential for over-treatment. Recently, Alderuccio et al. developed the Revised Mucosa-Associated Lymphoid Tissue International Prognostic Index, which aids in identifying patient groups at higher risk of early progression. Clinicians should utilize such tools while also considering the patient’s overall condition to determine the optimal treatment strategy [31]. Further large-scale studies are required to determine which patients can be managed safely with observation.

MALT lymphoma generally has a favorable long-term prognosis, but when it transforms into high-grade lymphoma, such as diffuse large B-cell lymphoma (DLBCL), the outcomes are poor [32]. However, no cases of DLBCL transformation were observed among the patients in our study. The reported frequency of MALT lymphoma transformation into DLBCL ranges from 2% to 8% [5,26]. However, existing reports on this transformation have primarily focused on MALT lymphomas in organs other than the colorectum. Research on DLBCL transformation in colorectal MALT lymphoma is limited, with only a few case reports available. Kang et al. reported one case of rectal MALT lymphoma transforming into DLBCL in South Korea [33]. The absence of DLBCL transformation in our study could be due to the relatively small sample size. Alternatively, it is possible that colorectal MALT lymphoma has an intrinsically lower tendency for transformation compared to MALT lymphoma in other organs. However, whether colorectal MALT lymphoma truly has a lower transformation rate remains uncertain, and further large-scale studies are necessary to clarify this issue. Among our patients, one individual died from disease progression. While we cannot exclude the possibility that this patient experienced transformation into DLBCL before death, confirmation was not possible. The patient’s condition deteriorated severely, leading to the cessation of further treatment and diagnostic evaluations, with only supportive care provided. This represents one of the inherent limitations of our retrospective study. In cases of disease progression in colorectal MALT lymphoma, it would be advisable for clinicians to assess the potential transformation into DLBCL to develop treatment strategies.

Previous studies have shown that colorectal MALT lymphoma can present as single or multiple lesions and exhibit various forms, such as flat, elevated, semipedunculated, or polypoid. Erosion or ulceration may also be observed in some cases [14,15,16,34,35,36].

Yachida et al. classified the endoscopic features of colorectal lymphoma into five types: polypoid, ulcerative, multiple lymphomatous polyposis, diffuse, and mixed/other. However, this classification considered various types of colorectal lymphomas together [37]. Jeon et al. categorized the endoscopic features of colorectal MALT lymphoma into four types: polyposis, subepithelial tumor, epithelial mass, and ileitis [10]. Additionally, in the case series by Tannoury et al., nine cases were classified as polypoid non-ulcerated lesions, large exophytic masses, and pseudo-tumoral enlarged folds [7]. Based on these previous studies, colorectal MALT lymphoma lesions can be classified into four major features: small polypoid lesions, large prominent masses, laterally spreading lesions, and inflammatory lesions. Therefore, in our study, we classified all lesions into four endoscopic types: polyposis, mass-forming, subepithelial lesion, and inflammatory. While our classification is similar to Jeon et al.’s, there are slight differences in the definitions of each type [10]. The polyposis type was the most prevalent in our cohort, which aligns with findings from a previously published case series and a literature review reporting polypoid lesions as the most common type [25,36]. However, in a retrospective study by Jeon et al., the polyposis type accounted for only 20% of cases, differing from our results [10]. Despite the variability in endoscopic findings, prior studies suggest that combining additional endoscopic features such as redness, vascular patterns, and lesion locations could allow clinicians to suspect colorectal MALT lymphoma before histological confirmation [5]. Of all cases in our study, 52.9% showed a first impression of lymphoma. The experience of the endoscopist and prior training on the endoscopic features of colorectal MALT lymphoma are thought to significantly impact diagnostic accuracy.

Our study has several limitations. First, its retrospective design introduces the potential for bias. Additionally, the relatively small number of patients included in the study limits the statistical power, which may constrain the generalizability of the findings. The study was conducted solely in hospitals located in specific regions of South Korea (Busan, Ulsan, and Gyeongnam), making it challenging to directly apply the results to other countries or regions.

Despite these limitations, this study holds significance as the first multicenter study on colorectal MALT lymphoma. Compared to previous studies, primarily limited to case reports or single-center studies, it offers more comprehensive results. Notably, our research highlights the excellent long-term prognosis of colorectal MALT lymphoma and offers clinically meaningful information to guide the diagnosis and treatment of this disease for medical practitioners.

## 5. Conclusions

This multicenter study highlights the indolent nature and excellent long-term prognosis of colorectal MALT lymphoma, with high survival rates. No significant associations were found between prognosis and endoscopic type or treatment modality. Observation may be a feasible alternative option in selected cases, particularly in those where treatment is not possible. Future large-scale studies are needed to confirm these findings and guide optimal management strategies.

## Figures and Tables

**Table 1:** Clinical characteristics at diagnosis.

Clinical Characteristic	Value
Age, years (IQR)	59 (48–69)
Sex, *n* (%)	
Male	27 (52.9)
Female	24 (47.1)
Underlying disease, *n* (%)	
None	47 (92.2)
Liver cirrhosis	1 (2.0)
Rheumatoid arthritis	1 (2.0)
Pulmonary tuberculosis	1 (2.0)
Pituitary adenoma	1 (2.0)
B symptom, *n* (%)	
No	50 (98.0)
Yes	1 (2.0)
LDH elevation, *n* (%)	8 (15.7)
B2MG elevation, *n* (%)	18 (35.3)
Initial stage, *n* (%)	
I	40 (78.4)
II	2 (3.9)
IIE	4 (7.8)
IV	5 (9.8) *
*Helicobacter pylori* infection, *n* (%)	
No	17 (33.3)
Yes	9 (17.6)
Not evaluated	25 (49.0)

IQR, interquartile range; LDH, lactate dehydrogenase; B2MG, beta-2-microglobulin; GI, gastrointestinal. * Two patients had bone marrow involvement.

**Table 2:** Endoscopic findings and treatment methods at diagnosis.

Endoscopic Findings and Treatment Methods	Value
Location, *n* (%)	
Terminal ileum	5 (9.8)
Cecum	4 (7.8)
Ascending colon	5 (9.8)
Transverse colon	2 (3.9)
Descending colon	4 (7.8)
Sigmoid colon	1 (2.0)
Rectum	20 (39.2)
Multiple sites	10 (19.6)
Multiplicity, *n* (%) *	
Single	16 (33.3)
Multiple	32 (66.7)
Endoscopic type, *n* (%)	
Polyposis	20 (39.2)
Mass-forming	13 (25.5)
Subepithelial lesion	15 (29.4)
Inflammatory	3 (5.9)
First endoscopic impression, *n* (%)	
Lymphoma	27 (52.9)
Colon polyp	13 (25.5)
Colon cancer	1 (2.0)
Neuroendocrine tumor	6 (11.8)
Lymphoid follicle	2 (3.9)
Others	2 (3.9)
Ulcerative colitis	1
Tuberculosis colitis	1
Treatment method, *n* (%)	
Endoscopic resection	16 (31.4)
Surgical resection	1 (2.0)
Radiation therapy	5 (9.8)
Chemotherapy	16 (31.4)
Observation	9 (17.6)
Combination treatment	4 (7.8)
Endoscopic resection + CTx	2
Endoscopic resection + RTx	2

CTx, chemotherapy; RTx, radiation therapy. * The inflammatory type was excluded.

**Table 3:** Initial stage and progression by endoscopic type.

	Endoscopic Type				*p*-Value
	Polyposis	Mass-Forming	Subepithelial Lesion	Inflammatory	
Initial stage					0.136
I	17	8	14	1	
II	1	1	0	0	
IIE	1	2	0	1	
IV	1	2	1	1	
Progression					0.813
No	18	13	14	3	
Yes	2	0	1	0	

**Table 4:** Disease progression by treatment method.

		Progression		*p*-Value
		No	Yes	
Treatment method	Endoscopic resection	15	1	0.889
	Surgical resection	1	0	
	Radiation therapy	5	0	
	Chemotherapy	14	2	
	Observation	9	0	
	Combination therapy	4	0	

**Table 5:** Clinical characteristics of patients who experienced progression despite treatment.

	Patient 1	Patient 2	Patient 3
Age/sex	65/F	63/M	73/M
Initial stage	I	I	I
B2MG elevation	Yes	Yes	Yes
LDH elevation	No	No	No
*Helicobacter pylori* infection	Positive	Negative	Negative
Endoscopic type	Polyposis type	Polyposis type	Subepithelial lesion type
Location	Rectum	Transverse colon	Descending colon
Initial treatment method	Chemotherapy (R-CVP)	Chemotherapy (R-CVP)	Endoscopic resection
Clinical course after initial treatment	Complete remission → Relapse (33 months later) → 2nd line chemotherapy (R-Benda) → Discontinuation of chemotherapy due to poor general condition → Observation → Death	Progression disease (41 months later) → 2nd line chemotherapy (R-Benda) → Stable disease → Transition to observation upon patient’s request → Stable disease maintained	Complete remission → Relapse (10 months later) → Chemotherapy (R-CVP) → Complete remission

LDH, lactate dehydrogenase; B2MG, beta-2-microglobulin; R-CVP, rituximab, cyclophosphamide, vincristine, prednisone; R-Benda, rituximab, bendamustine.

## Data Availability

Data are available from the corresponding author upon reasonable request.

## Supplementary Materials

The following supporting information can be downloaded at: https://www.mdpi.com/article/10.3390/cancers17050750/s1, Table S1. Clinical characteristics of patients who underwent observation without specific treatment.
