# Supplementary material for: Clinical Characteristics and Long-Term Prognosis of Colorectal Mucosa-Associated Lymphoid Tissue Lymphoma According to the Endoscopic Classification and Treatment Modality: A Multicenter Study
Source: Cancers (Basel). 2025 Feb 22;17(5):750. doi: 10.3390/cancers17050750 (PMC11899670; doi:10.3390/cancers17050750)
Supplement: Supplementary file 1 [file cancers-17-00750-s001.zip › cancers-3445132-supplementary.pdf]

Table S1. Clinical characteristics of patients who underwent observation without specific treatment

Patient	Age/sex	Initial stage	B2MG elevation	LDH elevation	*H. pylori* infection	Endoscopic type	Location	Progression	Follow up period (Month)
1	63/F	I	Yes	No	Not evaluated	Mass-forming type	Terminal ileum	No	119
2	54/F	I	No	Yes	Negative	Mass-forming type	Rectum	No	114
3	43/M	IV	No	Yes	Not evaluated	Mass-forming type	Terminal ileum	No	72
4	47/M	I	No	No	Not evaluated	Polyposis type	Multiple sites	No	61
5	57/F	II	No	No	Not evaluated	Mass-forming type	Cecum	No	60
6	59/F	II	No	No	Not evaluated	Polyposis type	Rectum	No	89
7	66/F	I	No	No	Not evaluated	Inflammatory type	Multiple sites	No	38
8	76/F	I	No	No	Not evaluated	Polyposis type	Cecum	No	70
9	69/M	I	Yes	No	Negative	Polyposis type	Descending colon	No	56

B2MG, beta-2-microglobulin; LDH, lactate dehydrogenase; *H. pylori, Helicobacter pylori.*
